# Molecular profiling of advanced soft-tissue sarcomas: the MULTISARC randomized trial

**DOI:** 10.1186/s12885-021-08878-2

**Published:** 2021-11-05

**Authors:** Antoine Italiano, Derek Dinart, Isabelle Soubeyran, Carine Bellera, Hélène Espérou, Christelle Delmas, Noémie Mercier, Sabrina Albert, Ludivine Poignie, Anne Boland, Aurélien Bourdon, Damien Geneste, Quentin Cavaille, Yec’han Laizet, Emmanuel Khalifa, Céline Auzanneau, Barbara Squiban, Nathalène Truffaux, Robert Olaso, Zuzana Gerber, Cédrick Wallet, Antoine Bénard, Jean-Yves Blay, Pierre Laurent-Puig, Jean-François Deleuze, Carlo Lucchesi, Simone Mathoulin-Pelissier, the MULTISARC study group

**Affiliations:** 1 Department of Medical Oncology, Institut Bergonié, University of Bordeaux, INSERM, Unité ACTION U1218, Bordeaux, France; 2 University of Bordeaux, Inserm, Bordeaux Population Health Research Center, UMR 1219, CIC-EC 1401/EUCLID Clinical Trials Platform, Bordeaux, France; 3 Unité de pathologie moléculaire, Institut Bergonié, Bordeaux, France; 4 Department of Biopathology, Institut Bergonié, U1218, Bordeaux, France; 5 Clinical Research and Clinical Epidemiology Unit, Institut Bergonié, Bordeaux, France; 6 Inserm, Pôle de Recherche Clinique, 75013 Paris, France; 7 ANRS (France Recherche Nord&sud Sida-hiv Hépatites), Clinical Trial Safety and Public Health, Paris, France; 8 Université Paris-Saclay, CEA, Centre National de Recherche en Génomique Humaine, 91057 Evry, France; 9 U1218, Institut Bergonié, Institut national de la santé et de la recherche médicale, Bordeaux, France; 10 Bioinformatics unit, Institut Bergonié, Bordeaux, France; 11 CHU, Bordeaux, France; 12 Department of Medical Oncology, Centre Léon Bérard, Lyon, France; 13 Sorbonne Paris Cité, Paris Descartes University, Georges Pompidou European Hospital, Paris, France

**Keywords:** Next generation sequencing, Soft-tissue sarcomas, Umbrella, Biomarker-driven

## Abstract

**Background:**

Soft-tissue sarcomas (STS) represent a heterogeneous group of rare tumors including more than 70 different histological subtypes. High throughput molecular analysis (next generation sequencing exome [NGS]) is a unique opportunity to identify driver mutations that can change the usual one-size-fits-all treatment paradigm to a patient-driven therapeutic strategy. The primary objective of the MULTISARC trial is to assess whether NGS can be conducted for a large proportion of metastatic STS participants within a reasonable time, and, secondarily to determine whether a NGS-guided therapeutic strategy improves participant’s outcome.

**Methods:**

This is a randomized, multicentre, phase II/III trial inspired by the design of umbrella and biomarker-driven trials. The setting plans up to 17 investigational centres across France and the recruitment of 960 participants. Participants aged at least 18 years, with unresectable locally advanced and/or metastatic STS confirmed by the French sarcoma pathological reference network, are randomized according to 1:1 allocation ratio between the experimental arm “NGS” and the standard “No NGS”. NGS will be considered feasible if (i) NGS results are available and interpretable, and (ii) a report of exome sequencing including a clinical recommendation from a multidisciplinary tumor board is provided to investigators within 7 weeks from reception of the samples on the biopathological platform. A feasibility rate of more than 70% is expected (null hypothesis: 70% versus alternative hypothesis: 80%). In terms of care, participants randomized in “No NGS” arm and who fail treatment will be able to switch to the NGS arm at the request of the investigator.

**Discussion:**

The MULTISARC trial is a prospective study designed to provide high-level evidence to support the implementation of NGS in routine clinical practice for advanced STS participants, on a large scale**.**

**Trial registration:**

clinicaltrial.gov NCT03784014.

**Supplementary Information:**

The online version contains supplementary material available at 10.1186/s12885-021-08878-2.

## Background

### Background and rationale

Soft-tissue sarcomas (STS) represent a heterogeneous group of rare tumors including more than 70 different histological subtypes [1]. Forty to 50 % of STS patients will develop metastatic disease with limited subsequent therapeutic options [2]. Once metastases are detected, treatment is mainly based on palliative chemotherapy and median overall survival (OS) of patients in this setting is about 12 to 20 months [3]. It is generally acknowledged that the benefits from chemotherapy in these diseases have reached a plateau and that new therapeutic strategies are urgently needed [4]. Despite recent insights into sarcoma genetics, a driver genetic alteration that could represent a therapeutic target has been identified in only a minority of sarcoma subtypes such as gastrointestinal stroma tumors. This suggests that the genetic understanding of this group of malignancies remains incomplete. While genome-wide expression and copy number technologies have improved disease classification, novel sequencing approaches are likely to change our understanding of the sarcoma tumorigenesis. These advances have the potential to better apply targeted therapies, thus further personalizing cancer therapy. Many somatic mutations will be actionable, a term used to define a known link between the mutation and response (or not) to the increasing range of targeted therapies already available in the clinic or in development. Recent studies have shown that the use of new sequencing technologies allows one to identify actionable mutation in up to 67% of participants with advanced STS [5–7]. Sarcomas such as gastrointestinal stromal tumors and dermatofibrosarcoma protuberans demonstrated the power of linking genetic mutations to effective targeted therapy and thus, the potential benefit of personalized medicine. For example, Imatinib, a selective inhibitor of KIT and PDGFRA has significantly improved outcome in these indications [8, 9]. Even more recently, a B cell gene expression signature established thanks to RNA sequencing was shown to be predictive of response to checkpoint blockade in patients with STS [10]. In this context, high throughput molecular analysis (next generation sequencing exome [NGS]) is a unique opportunity for identifying driver mutations that can change the usual one-size-fits-all treatment paradigm to a patient-driven therapeutic strategy.

### 2025 French genomic medicine plan

In response to a request from the French Prime Minister to promote access to genetic profiling in France, the French National Alliance for Life Sciences and Health (Aviesan) presented in 2016, the 2025 French Genomic Medicine (FMG) initiative. One aim of this plan is to integrate genome sequencing into routine clinical practice as well as to develop a national field of genomic medicine in partnership with research and industry. The MULTISARC trial as one of the four pilot studies, coordinated and sponsored by Inserm (the French National Institute of Health and Medical Research) and Aviesan Cancer Institute was set up in this context.

### Objectives

MULTISARC is a non-blinded randomized multicentre trial assessing whether NGS can be conducted for a large proportion of metastatic STS participants within a reasonable timeframe.

A key secondary objective is to determine whether a NGS-guided therapeutic strategy improves participant’s outcome compared to a standard strategy involving the same treatment for all participants in terms of OS.

Other objectives include: assessment of efficacy of the NGS-guided therapeutic strategy in terms of additional endpoints (Progression Free Survival, antitumor activity …), assessment of efficacy and safety of each targeted treatment involved in the MULTISARC trial, and estimation of proportion of participants presenting a targetable genomic alteration.

This clinical trial protocol follows the Standard Protocol Items: Recommendations for Interventional Trials (SPIRIT) guidelines (see SPIRIT checklist in online supplemental files) and is registered in a public trials registry (clinicaltrial.gov NCT03784014 on December 21st, 2018).

### Trial design

The design of this phase II/III trial is directly inspired by the design of umbrella and biomarker-driven trials [11, 12] allowing one to compare a personalized treatment, i.e. NGS-guided to a one-size fits all approach.

Following first-line treatment, participants will be randomized according to a 1:1 ratio with one participants randomized in experimental Arm “NGS” (treatment strategy based on NGS [exome, RNASeq] results) and one participants randomized in standard Arm “No NGS” (treatment strategy not based on NGS). Randomization will be stratified by centre and number of metastatic sites (0–1 versus ≥2) (minimization).

At the end of first-line treatment, the tumor profile of participants included in the experimental arm “NGS” will be discussed within a multidisciplinary/molecular tumor board (MTB) which aims at discussing the genomic profiles and at providing a therapeutic decision for each participant. MTB is composed of at least one oncologist, one pathologist and one molecular biologist and meet once a week. Participants will receive personalized treatment, that is, targeted treatment adapted to the target highlighted through NGS. Participants in whom no targets are identified or with biological material not qualified for genetic profiling will receive standard treatment.

**Fig. 1:**
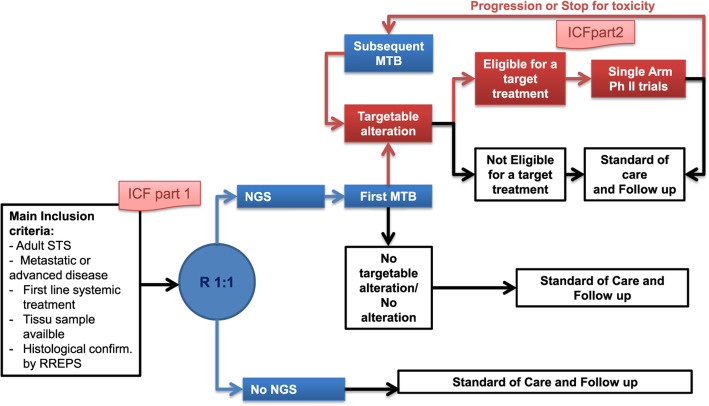
MULTISARC study design. STS: Soft-tissue Sarcoma; CT: Chemotherapy; RRePS: Pathological referral network for soft tissue and visceral sarcomas; ICF: Informed Consent Form; MTB: Molecular tumour board

Participants randomized in the standard arm “No NGS”, will be treated as per standard care. In case of exhaustion of available therapies, investigators will have the option to request a change of treatment arm in favour of the “NGS” arm. Crossover is only allowed from the “No NGS” arm to the “NGS” arm and requires certain specific conditions to be met (inclusion criteria …). The timing of the randomization is motivated by the situation of STS patients. There is currently no second standard treatment line for these patients. Therefore, the value of a direct comparison between the targeted therapies and a local standard therapy is not relevant. We want to investigate whether there is a significant difference in the survival of participants treated with a care strategy using NGS results compared to those treated with the local standard care strategy. The design of the study is presented in Fig. 1 in Appendix 1.

## Methods: participants, interventions, and outcomes

### Study setting

Up to 17 investigational centres of the French Sarcoma Group (Comprehensive Cancer Centres and university hospitals) across France will participate, with the recruitment of 960 participants. A 3-year accrual period with a 2-year fixed follow-up period is planned.

### Eligibility of the investigational centres and NGS platform

A first pre-selection was carried out and targeted the investigator centres that were the reference centres of the NETSARC network, located in metropolitan France and geographically covering the territory. The centres were subsequently selected on the basis of a feasibility survey (e.g. Availability of a technical platform for the preparation and dispatch of samples, human and logistics resources; Recruitment capacity …) and previous experience gained (e.g. Data entry deadlines, data quality …).

The biopathological platform (Institut Bergonié, Bordeaux) is labelled by the French National Cancer Institute (INCa), and ensures the qualification of samples, the extraction of nucleic acids and their quantification, and the shipping of nucleic acids to the Centre National de Recherche en Génétique Humaine (CNRGH-CEA), which is in charge of performing the sequencing. It also ensures the validation and interpretation of the genetic data after bioinformatics analyses of the raw data by the bioinformatics unit (Institut Bergonié, Bordeaux).

### Patient characteristics

Participants will provide written signed, informed consent (ICF) before any study procedures occur and prior to randomization. The eligible population consists of adults participants (aged ≥18 years) with unresectable locally advanced and/or metastatic STS confirmed by the French sarcoma pathological reference network (RRePS – ensuring a second expert pathological review of all suspected cases of soft tissue and visceral sarcomas).

Other eligibility criteria include absence of previous systemic treatment for advanced disease, 0–1 ECOG performance status, adequate hematological and metabolic functions (hemoglobin ≥9 g/dL, albumin ≥30 g/L), measurable disease according to RECIST v1.1 criteria [13], availability of suitable frozen archive tumor material and archived Formalin-Fixed Paraffin-Embedded block of specimen tumor sampling, and eligibility to first-line systemic treatment. Participants with radiological evidence of symptomatic or progressive brain metastases, or inability to swallow, or major problem with intestinal absorption are excluded from the study. The additional eligibility criteria are provided in Appendix 2.

### Interventions

All eligible participants will be randomized between the two investigated strategies before first-line treatment initiation. Participants will be treated with a first-line systemic treatment and tumor evaluation will be performed every 2 cycles during treatment.

For participants assigned to the “NGS” arm, high throughput molecular analysis (NGS whole exome and RNA sequencing) will be performed during the course of first-line treatment. At the end of it and irrespective of treatment outcome, participants will be managed according to the treatment strategy proposed by the MTB. Participants with a genomic alteration that may be associated with one of the study targeted treatments will be invited to participate in one single-arm phase II sub-trial involving the targeted treatment. Information will be provided together with a dedicated additional phase II trial inform consent to be signed by the participant. As in a conventional trial, participants must meet the specific eligibility criteria of the MULTISARC sub-trials before inclusion. Participants in treatment failure for the proposed treatment may be offered another sub-trial in agreement with the MTB decision if they present another genetic alteration that can be targeted by an available drug.

For participants assigned to the “No NGS” arm, they will be treated as per standard care. Both groups of participants will be followed up until death or study discontinuation whichever occurs first.

**Fig. 2:**
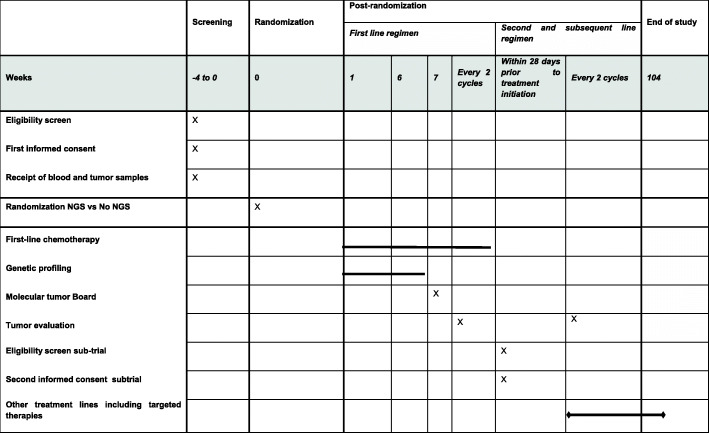
Schedule of enrolment, interventions, and assessments

A schedule of enrolment, assessment and intervention in accordance with SPIRIT guidelines is provided in Fig. 2 in Appendix 3.

### Outcomes/ endpoints

#### Feasibility and efficacy

NGS will be considered feasible if (i) NGS results are available and interpretable, and (ii) a report of exome sequencing including a clinical recommendation from a MTB is provided to investigators within 7 weeks from reception of the samples (blood and tumour samples) by the biopathological platform.

The design we use allows us to assess efficacy on two levels. The first level associated with randomization between the two arms corresponds to the assessment of treatment strategies (No NGS vs NGS). The second level, assessable only in the NGS arm, corresponds to the assessment of the targeted treatments per se. We will assess the efficacy of the treatment strategies based on OS and Progression Free Survival (PFS), defined as the delay from the date of randomization to the date of death (from any cause) or the date of progression as per RECIST v1.1 or death, whichever occurs first, respectively. Regarding the assessment of targeted therapies per se, we will assess their efficacy using best overall response defined as the best response recorded from date of targeted treatment initiation, 6-month non-progression rate defined as the rate of complete or partial response (CR, PR) or stable disease (SD) at 6 months, and objective response rate defined as the rate of CR or PR under targeted treatment. In addition we will also evaluate OS, PFS and change in tumor size from the date of targeted treatment initiation. All responses are evaluated according to RECIST v1.1. Safety of each treatment will be assessed as per NCI-CTCAE scale, version 5.

Regarding the proportion of targetable genomic alteration, participants for whom the MTB considers that at least one genetic alteration identified can be matched with one of the drugs available through the MULTISARC trial will be considered to present at least one targetable genomic alteration.

#### Cost-effectiveness

A micro-costing study will be conducted, in collaboration with the national network Inserm-RECaP of Research in Clinical Epidemiology and in Public Health, to estimate the real cost of NGS. Micro-costing studies collect detailed data on resources utilized (consumables, technician time, devices …) and the value of those resources.

The French National Health Insurance reimburses the vast majority of other resources involved in the care of participants in this trial. These costs will be extracted from the French National Health Insurance data. To do so, with the authorization of participants, we will gather their unique Health Insurance identification number. This number will enable us to merge the MULTISARC data with the National Health Insurance data of each included participant.

Some of the resources involved in the MULTISARC trial are not yet reimbursed by the French National Health Insurance as they are part of the innovative care strategy involving NGS. These resources will be gathered into the MULTISARC CRF and valorized by national conventional tariffs.

Utility scores will be assessed through the EuroQol-5D-5L self-questionnaire [14]. Participants will complete it each month during the first 6 months of follow-up, and every 6 month thereafter. A utility score between 0 (death) and 1 (perfect health status) will be computed and will allow to compute the quality adjusted life year (QALY) as the survival (in years) weighted by the utility score.

### Sample size

The sample size was calculated on the basis of the primary and the secondary objectives. We first estimated the sample size required to assess the superiority of the NGS-guided therapeutic strategy in terms of OS. We assumed a two-sided log-rank test, a 90% power, a 5% significance level, a 0.78 hazard ratio, an expected 24-month OS rate for the standard group of 16.9% (null hypothesis), a fixed 24-month follow-up period and a 1:1 allocation ratio. The standard arm involves about one third of patients who will not likely initiate a second line treatment, and two thirds who will. For this arm, we assumed a 9-month median OS. Overall, about 870 participants initiating 1st line treatment and with qualified material are therefore required. Accounting for 10% of participants without qualified material, 960 need to be included, leading to 480 participants in the experimental arm. Accounting for 90% of participants with qualified material, and then 90% with interpretable NGS results, 389 participants would have qualified and interpretable NGS results. This sample size will provide excellent power (> 99%) to test the hypothesis that the proportion of participants expected to present a validated report of exome sequencing including a clinical recommendation from the molecular tumor board within 7 weeks after reception of blood and tumor samples by one of the molecular platform is greater than 70% (primary objective: 70% (null hypothesis) versus 80% (alternative hypothesis).

### Molecular targets

To enable participants to access a large panel of innovative drugs, sponsor proposed to the scientific industrial community, the opportunity to participate to the MULTISARC program. To ensure transparency and equal treatment between the industrial actors who might be interested in participating in the program, a public call for partnership was launched. Thanks to this public-private partnership, about 10 targeted therapies will be included in the program. The Table 2 in Appendix 4 lists the main molecular alterations that will be targeted.

## Methods: data management, data monitoring and statistical methods

### Data management

Study data will be entered by the investigator or delegate using the electronic case report form developed via Ennov Clinical (version ≥7) software by the Data Manager of the study. Ennov Clinical software has been developed to meet the regulatory requirements. The Ennov Clinical software complies with the FDA requirements regarding IT systems used in clinical trial (“Guidance for Computerized systems Used in Clinical Trials”) as well as electronic signature (“21CFR part 11”) and various international norms. The Ennov company ensures the storage and maintenance of the database.

### Sequencing DATA

Sequencing data will be uploaded by the Centre National de Recherche en Génétique Humaine (CNRGH) on a secured server (CEA-TGCC, Bruyères Le Chätel), for quality control analysis and temporary storage. The bioinformatics unit located at Institut Bergonié will then electronically transfer the sequencing data via Secure FTP in order to analyze them on their computing cluster (HTC server). The biological and clinical interpretation report (MTB report) will be produced through GenVarXplorer software (GVX).

A pseudonymised .pdf copy of the biological report and of the MTB report will be electronically and securely transmitted via the case report form (CRF) to the investigation centres.

The sequencing data will be stored temporarily on the Institut Bergonié’s Data server (data replicated in 2 IT rooms for safety reasons). The virtual machine hosting the GVX software will be daily saved.

The 2025 FGMP provides for the provision of the Collecteur Analyseur de Données (CEA-CAD), an approved “healthcare data host” entity, which will allow the long-term archiving of sequencing data and potential further data processing.

### Statistical methods

Table 1 provides a summary of outcomes that will be measured and methods of analysis that will be carried out.

**Table 1:** Outcomes, measures and methods of analysis

Outcome	Hypothesis/outcome measure	Methods of analysis
**Primary outcome**		
Feasibility of NGS	80% of patients will present a validated sequencing report including a clinical recommendation from the MTB within 7 weeks from the receipt of the blood and tumor samples	Binomial test/ Confidence Interval
**Main secondary outcomes**		
Overall survival (OS)	Improvement of OS in NGS arm	Kaplan-Meir/Log-rank test
Progression-free survival (PFS)	Improvement of PFS in NGS arm	Kaplan-Meir/Log-rank test
Patients with targetable genomic alterations	Count and proportion of patients	Confidence Interval
Antitumor activity under targeted therapy (objective response, best overall response, disease control rate)	Count and proportion of patients following RECIST1.1 criteria.	Confidence Interval

### Governance structure

The Trials Steering Committee (TSC) brings together a variety of disciplines: representatives of sponsor, biological platform, CNRGH, pharmacovigilance, clinicians, statisticians and others collaborators. The TSC is responsible for the overall supervision of the trial. On behalf of the sponsor, the TSC ensures the proper conduct of the trial since the drafting of protocols, study design, continuation or termination (final or temporary) of the trial, monitoring of the trial, compliance with ethical principles, reviews of activity reports from other committees and gives its approval before any publication.

The Trial Management Team (TMT) composed of the operational staff involved in the MULTISARC trial (a representative of the sponsor, the coordinating investigator, the project manager, a clinical research associate, a statistician, a data manager, the biology project manager and a pharmacovigilance department representative) meets once a month and aims to ensure the daily management of the trial and to provide regular activity reports.

An Independent Data Monitoring Committee (IDMC) has been established to regularly assess the progress of the clinical trial, safety data, and critical efficacy endpoints, and to recommend to the Sponsor whether to continue, modify, or stop the trial. The IDMC is composed of a surgical oncologist, a medical oncologist, a statistician, a pharmacovigilance representative, an onco-geneticist and an ethicist.

An international Scientific Advisory Board (SAB) was also appointed by the sponsor. The SAB is responsible for: advising the TSC on the scientific and strategic relevance of the program, reviewing the objectives and design of the protocols, advising the TSC on drugs selection, evaluating sub-studies proposals and evaluating trial development and monitoring. The SAB is composed of a medical oncologist specializing in sarcoma, a medical oncologist specializing in digestive tumors, a statistician, a genomic specialist and representatives of patients’ associations.

## Ethics and dissemination

### Research ethics approval

The research will be carried-out with respect to the French regulation in effect. Prior to initiation, the protocol, informed consent forms will be submitted to and approved by an ethics committee (EC) and the Inserm college of reviewers. MULTISARC trial was granted approval by local Ethics Committee on October 8th, 2018, and authorized by the French authorities (ANSM) on September 25th, 2018.

Research and organization of the clinical trial were approved by the Comité National Informatique et Liberté (CNIL, National Committee of Informatics and Freedom). In accordance with regulatory requirements, a privacy impact assessment was also carried out and approved.

### Participant identification

In accordance with the legislative provisions in force and the RGPD regulations, persons having direct access to source data shall take all necessary precautions to ensure the confidentiality of information relating to persons who lend themselves to research.

The data collected in the MULTISARC CRF will be pseudonymised and will under no circumstances show the names of the persons concerned in clear text.

A unique identification code per participant will be constituted.

A paper correspondence form, containing the identity of the participant (surname, first name, date of birth), certain data relating to the study (inclusion date) and its identification code, will be kept by each investigating centre in the investigator’s file and archived for 15 years. These documents will be archived in a dedicated, locked room, with restricted access only to duly authorized persons, under the responsibility of the investigator centre.

### Protocol amendments

Any substantial modification, i.e. any modification which may have a significant impact on the safety of participants, on the conditions of validity and results of the study, on the quality and safety of investigational products, on the interpretation of scientific documents that support the study or the procedures of this, are the subject of a written amendment that is submitted to the trial sponsor; the latter must obtain, before its implementation, approval from the EC and authorization from the Relevant Authority.

All amendments are validated by the Sponsor, and by all the parties involved with the study concerned by the modification, before submission to the EC and the competent authority. This validation may require a meeting of the Steering Committee and/or the Independent Data Monitoring Committee. All protocol amendments must be made known to all investigators participating in the study. The investigators undertake to comply with its contents.

All amendment that modifies participant care or the benefits, risks and constraints of the study, are the subject of a new participant information letter and a new consent form, the obtainment of which follows the aforementioned procedure.

### Consent

The consent of participants is obtained by signing an informed consent form. This paper form is given to the participant by the investigating physician during the consultation to present the MULTISARC trial. The signature takes place after oral information by the investigator, combined with the reading of the information notice detailing the study, and in particular the processing of personal data. A system of checkboxes allows participants to clearly understand the scope of the agreement they give for the processing of their data and for the secondary use of data and samples.

Participants included in the MULTISARC trial, randomized in Arm NGS and for which NGS molecular profile has shown at least one genomic alteration that can be targeted by a therapy available in one of the MULTISARC sub-trials, will be invited to participate in this sub-trial. After information has been given and participant has agreed to participate, both participant and investigator will sign a second informed consent form of the specific MULTISARC sub-trial (ICF2).

## Conclusions/discussion- challenges and benefits

The MULTISARC trial is the first randomized precision medicine studies ever conducted in STS participants. Its design aims to provide high-level evidence to support the implementation of NGS in routine clinical practice for advanced STS participants, on a large scale. MULTISARC will include 960 participants who will be followed from the diagnosis of the metastatic disease to death with the possibility to receive multiple treatment lines including targeted therapies based on the NGS. This study represents a unique opportunity to both measure the time frame needed to analyze and interpret sequencing data, to improve the quality of diagnosis and therapeutic orientation and to understand the biological mechanisms involved in the sensitivity and resistance to anti-cancer therapies in the metastatic setting.

The biomarker-strategy design is useful in situations where it is either not feasible or ethical to test the biomarker in the entire population due to logistical reasons (financial issues). The design is also useful when we want to test whether the overall treatment effect based on the biomarker-based strategy approach is superior to that of the standard strategy. In such a context, the expected results are related to the prevalence of biomarker-positive participants in NGS arm, as the presence of biomarker-negative participants in both strategy arms dilute the treatment effect. In case of efficacy of the biomarker-based strategy, the evaluation of targeted therapies in parallel sub-trials independently determines whether a clinical benefit is visible for each biomarker-drug combination.

The number of molecules under investigation and therefore the number of experimental arms has not been fixed in advance. The opening of a clinical trial with several treatment arms is challenging. The design will allow us to add new research comparisons if compelling clinical and scientific research questions arise. The proper conduct of such a study requires anticipation. CRF need to be appropriate to the comparisons and allow additions and updates and, as far as possible, should not impact data quality. The establishment of new contracts with industrial partners and drug supply logistics are things that must be coordinated in parallel with the management of active experimental arms [15, 16].

MULTISARC is a unique project that has started accrual in 2019 and will bring its share of challenges. These would be impossible without the mobilization and investment of professionals from several trades and disciplines (Oncologists, Oncogeneticists, Methodologists, Project leaders, Biostatisticians, Bioinformaticians, Biologists, Data-managers, Clinical research assistants, ethicists), and patients.

### Supplementary Information

**Additional file 1.** SPIRIT Checklist: Recommended items to address in a clinical trial protocol and related documents.

**Additional file 2.** List of members of the MULTISARC study group.

## Data Availability

Not applicable.

## Appendix 2

### List of additional eligibility criteria

**Table 2:** List of additional eligibility criteria

N°	Inclusion criteria	Non-inclusion criteria
1	Archived FFPE block of specimen tumor sampling obtained anytime during disease development for research purpose,	Previous allogeneic bone marrow transplant,
2	No prior or concurrent malignant disease diagnosed or treated in the last two years before inclusion, except for in situ carcinoma of the cervix and adequately treated basal cell or squamous cell carcinoma of the skin and prostate cancer,	Evidence of severe or uncontrolled systemic disease (uncontrolled hypertension, active bleeding diatheses, or active Hepatitis B, C and HIV or active autoimmune disease),
3	Participant with a social security in compliance with the French law	Any condition which in the Investigator’s opinion makes it undesirable for the subject to participate in the trial or which would jeopardize compliance with the protocol,
4		Individuals deprived of liberty or placed under guardianship
5		Pregnant or breast feeding women,
6		Men or women refusing contraception,
7		Previous enrolment in the present study,
8		Any contraindication to first-line systemic treatment.

## Appendix 4

### List of available targeted therapies

**Table 3:** : list of available targeted therapies and corresponding mechanisms of action

Drugs	Molecular targets
Ceritinib	ALK
	ROS
Capmatinib	MET
Lapatinib	ERBB2
	EGFR
Nilotinib	KIT
	PDGFRA
	CSF1R
Trametinib	KRAS, NRAS, HRAS
	PTPN11
	NF1
	MAP 2 K
Trametinib + Dabrafenib	BRAF
Durvalumab + Olaparib	PDL1
	PARP
Palbociclib	CDK4
	CDK6
Glasdegib	SMO
TAS-120	FGFR

## Abbreviations

CNIL: Comité National Informatique et Liberté
CNRGH: Centre National de Recherche en Génétique Humaine
EC: Ethics committee
ICF: Informed consent form
IDMC: Independent Data Monitoring Committee
Inserm: Institut national de la santé et de la recherche médicale
MTB: Molecular tumor board
NGS: Next-generation sequencing
OS: Overall survival
RECaP: Research in Clinical Epidemiology and in Public Health
RECIST: Response evaluation criteria in solid tumors
RRePS: Réseau de Référence en Pathologie des Sarcomes des tissus mous et des viscères Sarcomes
SAB: Scientific Advisory Board
STS: Soft-tissue sarcoma
TSC: Trial Steering Committee
TMT: Trial Management Team
