# Supplementary material for: Molecular profiling of advanced soft-tissue sarcomas: the MULTISARC randomized trial
Source: BMC Cancer. 2021 Nov 5;21:1180. doi: 10.1186/s12885-021-08878-2 (PMC8570026; doi:10.1186/s12885-021-08878-2)
Supplement: Supplementary file 2 — Additional file 2. List of members of the MULTISARC study group. [file 12885_2021_8878_MOESM2_ESM.pdf]

MULTISARC study group

- **Executive Committee**

T. Aparicio, C. Bellera, G. Bloch, C. Chomienne, JF. Deleuze, H. Espérou, A. Eychène, A. Italiano, P. Laurent-Puig, F. Lethimonnier, Y. Levy, C. Levy-Marchal, FX. Mahon, S. Mathoulin-Pélissier, P. Michel, F. Nowak

- **Trial Steering Committee**

A. Italiano (Coordinating investigator), S. Albert, C. Bellera, V. Besson-Dubourg, A. Boland-Auge, M. Caralp, JF. Deleuze, C. Delmas, A. Diallo, D. Dinart, H. Espérou, L. Gauquelin, F. Geillon, A. Gelley, C. Girault, D. Gozlan, Y. Laizet, E. Landry, P. Laurent-Puig, F. Lethimonnier, C. Lucchesi, C. Malle, S. Mathoulin-Pélissier, N. Mercier, F. Nowak, C. Nunez, G. Perkins, L. Poignie, I. Soubeyran, B. Squiban, M. Treuil Peraldi, N. Truffaux, C. Wallet, B. Zafirova

- **International Scientific Advisory Board**

R. Maki (Chair), JP. Bertou, E. Campo, G. Folprecht, E. Lecointe-Artzner, R. Salazar, S. Stacchiotti, M. Redman

- **Trial Management Team**

A. Italiano (Chair), S. Albert, C. Bellera, A. Cantan, F. Cauet-Devillards, C. Delmas, D. Dinart, L. Gauquelin, A. Gelley, Q. Guillochon, D. Jean, S. Lemestre, C. Malle, C. Nunez, L. Poignie, N. Sabour, B. Squiban, M. Treuil Peraldi, B. Zafirova

Coordinating Unit

CIC-EC 1401/EUCLID: S. Albert, C. Bellera, A. Bénard, A. Cantan, D. Dinart, F. Cauet-Devillards, A. Gelley, Q. Guillochon, D. Jean, C. Nunez, S. Mathoulin-Pelissier, L. Poignie, L. Richert, C. Schwimmer, M. Treuil Peraldi, C. Wallet

Coordinating Pharmacy: A. Gelley, L. Poignie

Laboratory:

CEA, Centre National de Recherche en Génomique Humaine: JF. Deleuze (head), D. Bacq, C. Besse, A. Boland, B. Fin, Z. Gerber, V. Meyer, R. Olaso, MA. Palomares

Department of Biopathology, U1218, Institut Bergonié: I. Soubeyran (head), C. Auzanneau, M. Boucheix, L. Dureau, E. Khalifa, C. Ruysschaert, B. Squiban, N. Truffaux

Bioinformatics unit, Institut Bergonié: C. Lucchesi (head), A. Bourdon, Q. Cavaille, D. Geneste, Y. Laizet
